# A Loss-Of-Function Analysis Reveals That Endogenous Rem2 Promotes Functional Glutamatergic Synapse Formation and Restricts Dendritic Complexity

**DOI:** 10.1371/journal.pone.0074751

**Published:** 2013-08-26

**Authors:** Anna R. Moore, Amy E. Ghiretti, Suzanne Paradis

**Affiliations:** Department of Biology, National Center for Behavioral Genomics and Volen Center for Complex Systems, Brandeis University, Waltham, Massachusetts, United States of America; Dalhousie University, Canada

## Abstract

Rem2 is a member of the RGK family of small Ras-like GTPases whose expression and function is regulated by neuronal activity in the brain. A number of questions still remain as to the endogenous functions of Rem2 in neurons. RNAi-mediated Rem2 knockdown leads to an increase in dendritic complexity and a decrease in functional excitatory synapses, though a recent report challenged the specificity of Rem2-targeted RNAi reagents. In addition, overexpression in a number of cell types has shown that Rem2 can inhibit voltage-gated calcium channel (VGCC) function, while studies employing RNAi-mediated knockdown of Rem2 have failed to observe a corresponding enhancement of VGCC function. To further investigate these discrepancies and determine the endogenous function of Rem2, we took a comprehensive, loss-of-function approach utilizing two independent, validated Rem2-targeted shRNAs to analyze Rem2 function. We sought to investigate the consequence of endogenous Rem2 knockdown by focusing on the three reported functions of Rem2 in neurons: regulation of synapse formation, dendritic morphology, and voltage-gated calcium channels. We conclude that endogenous Rem2 is a positive regulator of functional, excitatory synapse development and a negative regulator of dendritic complexity. In addition, while we are unable to reach a definitive conclusion as to whether the regulation of VGCCs is an endogenous function of Rem2, our study reports important data regarding RNAi reagents for use in future investigation of this issue.

## Introduction

The RGK (Ras, Rem, Rem2, Gem/Kir) protein family is a subclass of small Ras-like GTPases structurally distinct from canonical GTPases. The major distinguishing features of the RGK protein family include: calmodulin and 14-3-3 protein binding sites, noncanonical amino acid substitutions in the guanine nucleotide binding domain, and a lack of identified guanine nucleotide exchange factors (GEFs) or GTPase activating proteins (GAPs) [1]. RGK proteins have been implicated in mediating cytoskeletal rearrangements [2–5] and inhibition of voltage-gated calcium channel currents [6–10]. Additionally, RGK proteins are differentially expressed in specific tissues with transcriptional regulation of their mRNA expression mediated by a variety of extrinsic factors (i.e. glucose, mitogens, and neuronal depolarization) [1,11–14]. For example, Rem2 is highly expressed in the brain [12] and is upregulated in response to neuronal depolarization [15].

Both loss-of-function and overexpression approaches have been used to try to determine the physiological function of Rem2. Overexpression studies have shown that Rem2 can inhibit voltage-gated calcium channel currents in a variety of cell types [8,16–19], as reported for other RGK proteins [20]. However, whether VGCC inhibition is due to interference with channel trafficking to the plasma membrane by RGK proteins [21] or RGK-mediated inactivation of channels already at the surface [8,16], remains controversial. Additionally, overexpression of Rem2 in COS cells causes increased membrane extensions [21], suggesting Rem2 may also be involved in cytoskeletal rearrangements. However, a major caveat of these studies is that they are based on overexpression of Rem2 protein.

To date, only a handful of studies have examined the effect of knockdown of endogenous Rem2 [15,18,22–25]. In cultured hippocampal neurons, both siRNA [15] and shRNA-mediated Rem2 [25] knockdown leads to a decrease in excitatory synapse density, as assayed by both immunocytochemistry and electrophysiology [15,25], and an increase in dendritic complexity, as assessed by Sholl analysis [25]. These phenotypes could be rescued by co-transfection with an RNAi-resistant Rem2 cDNA [25], suggesting that they are due specifically to Rem2 knockdown and not the result of off-target effects of the shRNA construct. Moreover, a separate loss-of-function study also observed decreased frequency of miniature excitatory postsynaptic currents (mEPSCs) upon Rem2 knockdown, consistent with a change in synapse density [18]. Further studies demonstrated that phosphorylation of Rem2 by CaMKII is required for Rem2-mediated suppression of dendritic complexity via Rem2 translocation to the nucleus [24]. Importantly, the mutation of key phosphorylation sites in the Rem2 coding sequence interferes with Rem2 nuclear localization and causes increased dendritic complexity, mimicking the Rem2 RNAi phenotype and further supporting the role of Rem2 as a negative regulator of dendritic complexity [24].

In addition to these neuronal phenotypes, knockdown of Rem2 in human embryonic stem cells (hESCs) inhibits the proliferative ability of hESCs via regulation of cyclin D1 localization and p53 transcriptional activation, which promote cell cycle progression and protection from apoptosis, respectively [23]. In a subsequent study, morpholino-mediated knockdown of Rem2 expression in zebrafish during embryonic development caused a loss of neural tissue and increased apoptosis in the nervous system [22]. Taken together, these two studies suggest a function for Rem2 during generation of the nervous system in regulating cellular proliferation and survival [22,23], in addition to the role of Rem2 in post-mitotic neurons [15,18,24,25].

Despite the data summarized above, the endogenous function of Rem2 in neurons remains unclear due to 1) the suggestion that shRNAs targeting Rem2 have confounding off-target effects [18], and 2) the absence of an effect on VGCC function upon Rem2 knockdown with these shRNAs [18]. In this study, we sought to determine the function of endogenous Rem2 by developing additional RNAi technology, a second independent shRNA targeting Rem2, to effectively decrease Rem2 expression and assay dendritic morphology, glutamatergic synapse formation, and VGCC current amplitude. To accomplish this goal, we first confirmed the efficacy of Rem2 knockdown with a second independent shRNA by immunocytochemistry using a Rem2 antibody. Next, we investigated the effects of the second Rem2 shRNA on dendritic morphology and excitatory synapse density by comparison to our previously validated shRNA [24,25]. We demonstrate that in agreement with previously published reports [24,25], we observe a significant increase in dendritic complexity and a significant decrease in excitatory synapse density by immunocytochemistry. Further, Rem2 knockdown affects functional excitatory synapse density, as shown by a decrease in mEPSC frequency. These effects, observed with multiple hairpins, are successfully rescued with RNAi-resistant Rem2 cDNAs. Lastly, we examined VGCC current amplitude and find that while we can recapitulate the decrease in calcium current previously observed with Rem2 overexpression, Rem2 knockdown with only one of the two shRNAs used in this study demonstrates the predicted phenotype on VGCC current upon Rem2 knockdown: an increase in calcium current amplitude. This result leaves us unable to reach a conclusion as to whether or not endogenous Rem2 regulates VGCC function, but provides new information about the usefulness of these RNAi tools for investigating the role of Rem2 in mediating calcium influx through VGCCs.

## Methods

### Ethics Statement

Animals were handled according to the National Institutes of Health Guide for the Care and Use of Laboratory Animals and approved by the Brandeis University Animal Care and Use Committee.

### 293T cell culture and Western Blotting

HEK 293T cells were grown in 12-well tissue culture plates. The cells were transfected by the calcium phosphate method [26] with 500 ng GFP, 100 ng myc-CMV Rem2 or myc-CMV RNAi-resistant Rem2 cDNAs, and 1 µg of either shRNA 1 (RNAi 1) or shRNA 2 (RNAi 2) targeting Rem2. The RNAi-resistant Rem2 cDNA is not susceptible to knock down by either RNAi 1 or RNAi 2. It was generated from a wildtype myc-tagged Rem2 cDNA using site-directed mutagenesis to introduce silent point mutations into the regions of the Rem2 coding sequence recognized by each shRNA. The forward mutagenesis primers used are listed below, with mutations indicated in lower case: Resistant to RNAi 1: GACACCTATGAGAGgCGaATtATGGTGGACAAAGAA; Resistant to RNAi 2: GCTCACGAGATGGAaAAtTCcGAGGACACCTATG. For Western blotting, the HEK 293T cells were lysed in Sample Buffer (SDS, bromphenol blue, 1*M* Tris pH 6.8, glycerol), and proteins were separated by electrophoresis on a 10% SDS–PAGE gel. The proteins were transferred to a nitrocellulose membrane, which was then blocked in a 5% milk/1× TBST solution for 1 h, then incubated overnight at 4°C in primary antibody [anti-Rem2 (1:100 [25]) and anti-β-actin (Abcam; 1:1000). The following day, the membrane was washed two times for 20 min in 1× TBST, and incubated in the appropriate secondary antibody (Licor; 1:5000) for 2 h at room temperature. Following two 20-min washes in 1× TBST, the blot was developed using the Odyssey Western Blotting system (Licor).

### Neuronal Cell Culture and Transfection

Hippocampal neurons were cultured on a confluent astrocyte feeder layer. Astrocytes and neuronal cultures were prepared as previously described [25]. Briefly, astrocytes were isolated from P0 rat cortex and plated at low density on 12-cm glass coverslips coated with poly-D-lysine (20 µg/mL) and laminin (3.4 µg/mL) in 24-well plates. Hippocampi from E18 rat pups were dissociated and cultured at a density of approximately 80,000 neurons/well in Neurobasal media with B27 supplement (Invitrogen). AraC (Sigma) was added to a final concentration of 5 µM 24 hours after plating.

Neurons were transfected at 4 DIV using the calcium phosphate method [26], providing a relatively low transfection efficiency (~10%) and thus allowing a transfected neuron to develop in an otherwise unaltered network. All neurons were co-transfected with 500 ng of CMV-GFP plasmid/well plus: empty pSuper vector at 133 ng/well (mock), or pSuper-shRNA plasmids containing an shRNA against Rem2 at 33 ng (RNAi 1) or 15 ng (RNAi 2)/well (RNAi conditions). For overexpression conditions, the RNAi-resistant Rem2 cDNA was transfected at 100 ng/well. For rescue conditions, GFP and shRNA and RNAi-resistant Rem2 cDNA were co-transfected at above concentrations. The co-transfection efficiency of up to three plasmids is 93% [24,25].

### Immunocytochemistry

Neurons were fixed and stained at 14 DIV as previously reported [25]. For the synapse density assay, neurons were incubated overnight in a humidified chamber with mouse anti-PSD-95 (1:500: UC, Davis/NIH NeuroMab Facility) and rabbit anti-synapsin (1:1000; Millipore). For the Rem2 expression experiments, neurons were incubated in rabbit anti-Rem2 (1:100 [25]) and mouse anti-MAP2 (1:1000; Sigma). All antibody dilutions were prepared in 1X GDB (0.1% gelatin, 0.3% Triton X-100, 4.2% 0.4M phosphate buffer, and 9% 5M NaCl). Following overnight incubation, coverslips were washed 3 times in 1X PBS and incubated with the appropriate Cy3- and Cy5-conjugated secondary antibodies (1:500; Jackson ImmunoResearch Laboratories) in 1X GDB for 2 hours at room temperature. Coverslips were again washed 3 times in 1X PBS and mounted on glass slides with Aquamount (Lerner Laboratories).

### Electrophysiology

Electrophysiological recordings were performed at 8 DIV (VGCC current recordings) and 14 DIV (mEPSC recordings), 4 and 10 days after transfection, respectively. Whole-cell voltage clamp recordings were obtained at 34°C from GFP-positive cells with a Multiclamp 700B amplifier and a Digidata 1440A digitizer controlled by pClamp10 software. R_s_ and R_IN_ were monitored throughout experiments using Lab Bench (Clampex 10.2) and cells that exhibited changes of more than 20% in either of these parameters throughout the course of the recording were discarded. For voltage-gated calcium channel (VGCC) current recordings the extracellular solution contained (in mM): 125 NaCl, 26 NaHCO_3_, 2.3 KCl, 1.26 KH_2_PO_4_, 2 CaCl_2_, 2 MgSO_4_, 10 glucose, 1 µM tetrodotoxin (TTX; Abcam Biochemicals), 10 µM ZD7288 (Sigma), 15 mM TEA (Sigma), 5 mM 4-AP (Sigma), and 5 mM CsCl (Sigma). Patch pipettes (3-5 MΩ) were filled with intracellular solution containing (in mM): 120 Cesium Methanesulfonate, 10 CsCl, 10 HEPES, 0.5 EGTA, 2 MgSO_4_, 3 Na _2_ATP, and 0.3 Na _2_GTP, 10 mM Phosphocreatine, and 1 mM QX-314 (pH 7.3, adjusted with CsOH). For the I–V curves VGCC currents were induced by a 300 ms step from -90 to + 70 mV in 5 mV steps from a holding potential of -70 mV. For measuring peak calcium current amplitude cells were given a ramp step from -80 mV to +50 mV over 500 ms. Calcium currents were measured offline in Clampfit 10.2 (Molecular Devices).

For the mEPSC recordings the external solution contained (in mM): 125 NaCl, 26 NaHCO_3_, 2.3 KCl, 1.26 KH_2_PO_4_, 2 CaCl_2_, 2 MgSO_4_, 10 glucose, 1 µM TTX and 20 µM Picrotoxin (Sigma). The internal pipette solution contained (in mM): 120 Cesium Methanesulfonate, 10 CsCl, 10 HEPES, 0.5 EGTA, 2 MgSO_4_, 3 Na _2_ATP, and 0.3 Na _2_GTP, and 10 mM Phosphocreatine (pH 7.3, adjusted with CsOH). To record mEPSCs, the membrane potential was held at -70mV and events were filtered at 1 kHz. Data was recorded in 3 epochs at 100 s each for a total duration of 300s per cell. mEPSCs were then evaluated offline in Clampfit 10.2 (Molecular Devices).

Data is presented as mean ± S.E.M and statistical significance was calculated using a one-way ANOVA and student’s independent *t*-Test. For the cumulative distribution plots 100 mEPSC amplitudes were randomly selected for each cell. Statistical significance for the cumulative distribution plots was calculated using a Kolmogorov-Smirnov test.

### Image acquisition and analysis

All image acquisition and quantification were performed in a blinded manner. Twelve-bit images were acquired on an Olympus Fluoview300 confocal microscope using a 60X (for immunocytochemical analysis) or 20X (for dendritic complexity analysis) oil objective. Images were acquired as a z-stack (5-15 optical sections and 0.5 µm (60X) or 1 µm (20X) step size), and maximum intensity projections were created from the stacks.

For Rem2/MAP2 expression experiments, staining intensity was quantified using MetaMorph imaging analysis software. A 25 µm stretch of dendrite was identified as the region of interest using the GFP channel as a guide. This region was then copied to the Rem2 and MAP2 channels, and the average pixel intensity of staining in the region was determined. Approximately 15-20 images were acquired and analyzed for each condition from at least 2 separate coverslips for a total of three experiments. The staining intensity for each condition was calculated by averaging the intensities for each image across all conditions, and significant differences between conditions were determined via a two-way ANOVA followed by a Tukey’s post hoc test for significance.

For measuring dendritic complexity, Sholl analysis was performed by applying a series of 11 concentric circles of increasing radii (10 µm intervals) drawn around the center of the cell body using the ImageJ plugin Concentric Circles (NIH). The number of dendrites crossing at each radius for each condition was calculated by averaging the number of crossings from every image. For each experiment 10-20 images from two separate coverslips were acquired and analyzed for each condition for a total of three experiments. Significance was determined using a two-way multivariate ANOVA (factors were transfection group and circle radius) followed by a Tukey’s post hoc test for significance.

For PSD-95/synapsin I experiments, synapse density was quantified as the overlap of GFP, anti-PSD-95, and anti-synapsin I staining using MetaMorph image analysis software [25]. For each experiment, the threshold for the PSD-95 and synapsin I channels was determined visually using an image of a control neuron and chosen such that all punctate structures would be included in the analysis. Approximately 15-20 images from at least two separate coverslips were acquired and analyzed for each condition within an experiment for a total of three experiments. Synapse density values within each experiment were normalized to account for the variation in antibody staining and neuron density across experiments (see 15 for details). Statistical analysis was performed comparing each experimental condition to control on the combined raw data from all experiments using a two-way between-effect ANOVA followed by a Tukey’s post hoc test for significance. Error bars denote standard error of the mean.

## Results

**Figure 1:**
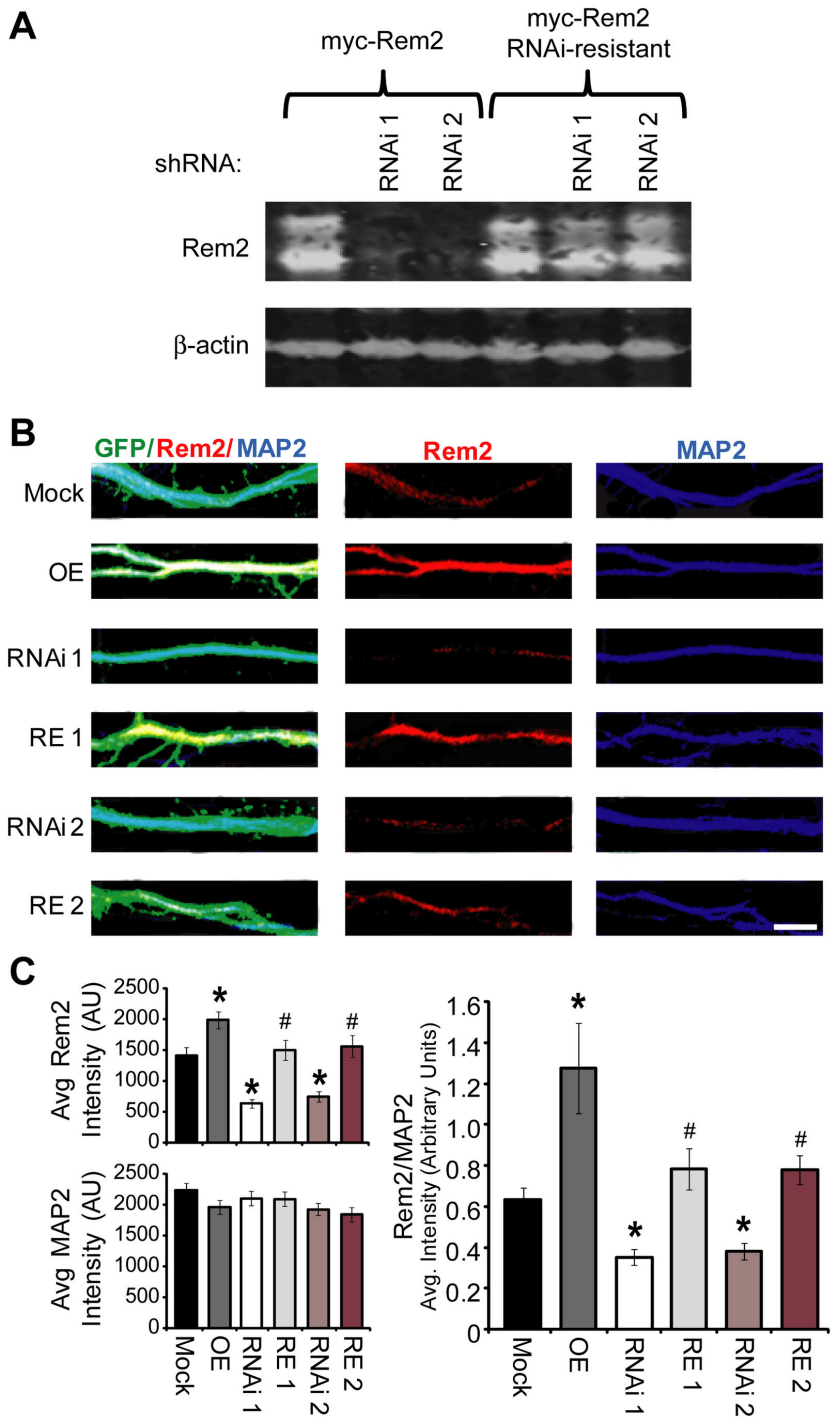
Rem2 expression in hippocampal neurons. **A**) Western blot with anti-Rem2 antibody of HEK 293T cell lysates. anti-β-actin serves as a loading control. HEK 293T cells were transfected with either myc-Rem2 or RNAi-resistant myc-Rem2 and either the Rem2 short hairpin 1 (RNAi 1) or the Rem2 short hairpin 2 (RNAi 2) to confirm Rem2 knockdown and rescue. **B**) Representative images of Rem2 (red) and MAP2 (blue) staining in neurons transfected with GFP and either Mock (empty pSuper vector), overexpression with the Rem2 RNAi-resistant cDNA (OE), RNAi with the Rem2 short hairpin 1 (RNAi 1), RNAi with a second Rem2 short hairpin (RNAi 2), or rescue of RNAi 1 (RE 1), or RNAi 2 (RE 2) with the Rem2 RNAi-resistant cDNA. Scale bar = 5 µM. **C**) Average Rem2 staining intensity (top left) or MAP2 intensity (bottom left) measured in arbitrary units for each condition (Right). Average Rem2 immunostaining intensity normalized to MAP2 intensity in hippocampal neurons. n > 40 neurons per condition. * p ≤ 0.05 compared to mock or ^#^ p ≤ 0.05 compared to the appropriate RNAi condition (i.e. RE1 vs. RNAi 1) using a two-way ANOVA followed by a Tukey’s post hoc test.

Using a rabbit polyclonal antibody raised against purified GST-tagged rat Rem2, we previously demonstrated that a short hairpin targeting Rem2 (in this report referred to as RNAi 1) decreases Rem2 protein levels by about 70% in cultured hippocampal neurons [25]. Using this same antibody, we sought to determine if a second, entirely independent shRNA (RNAi 2) could similarly decrease Rem2 protein levels. We first confirmed that the second hairpin construct could sufficiently knock down Rem2 and that the RNAi-resistant cDNA, which had been engineered to be resistant to both shRNAs, was in fact resistant to RNAi 2 by Western blotting of heterologous cell lysate. We co-transfected either wildtype myc-tagged Rem2 cDNA or RNAi-resistant Rem2 cDNA along with either the Rem2 RNAi 1 as a control or RNAi 2 (Figure 1A) in HEK 293T cells. Similarly to RNAi 1, RNAi 2 significantly decreased wildtype Rem2 protein expression on a Western blot, while the level of RNAi-resistant Rem2 protein was not affected by co-transfection with either RNAi 1 or RNAi 2 (Figure 1A).

After verifying the efficacy of this second independent hairpin in HEK 293T cells, we proceeded to investigate the ability of RNAi 2 to affect Rem2 expression in neurons. To this end, cultured hippocampal neurons were co-transfected at 4 DIV with GFP and either an empty pSuper vector (“Mock”), an shRNA construct targeting Rem2 (“RNAi 1”), a second independent shRNA against Rem2 (“RNAi 2”), an RNAi resistant Rem2 cDNA (overexpression, “OE”), RNAi 1 and an RNAi-resistant Rem2 cDNA (the rescue condition, “RE 1”), or RNAi 2 and an RNAi-resistant cDNA (also a rescue condition, “RE 2”). We have previously verified that the triple co-transfection efficiency in our neuronal cultures is greater than 90% [24,25]. Cultures were fixed and stained at 14-15 DIV with antibodies that recognize Rem2 and MAP2 (microtubule-associated protein 2) as a control and imaged on a confocal microscope (Figure 1B).

We found that the average intensity of the Rem2 staining decreased by approximately 70% in neurons that had been transfected with RNAi 1 or RNAi 2 while the average intensity of Rem2 staining was increased in the OE condition compared to mock transfection (Figure 1B, C). The decreased intensity of Rem2 immunostaining in either the RNAi 1 or RNAi 2 condition was completely rescued by co-expression with a Rem2 RNAi-resistant cDNA (Figure 1B, C). As expected, the intensity of the MAP2 staining was unchanged (Figure 1B, C). These data demonstrate the efficacy and specificity of the RNAi 2 construct in decreasing Rem2 levels in hippocampal neurons.

**Figure 2:**
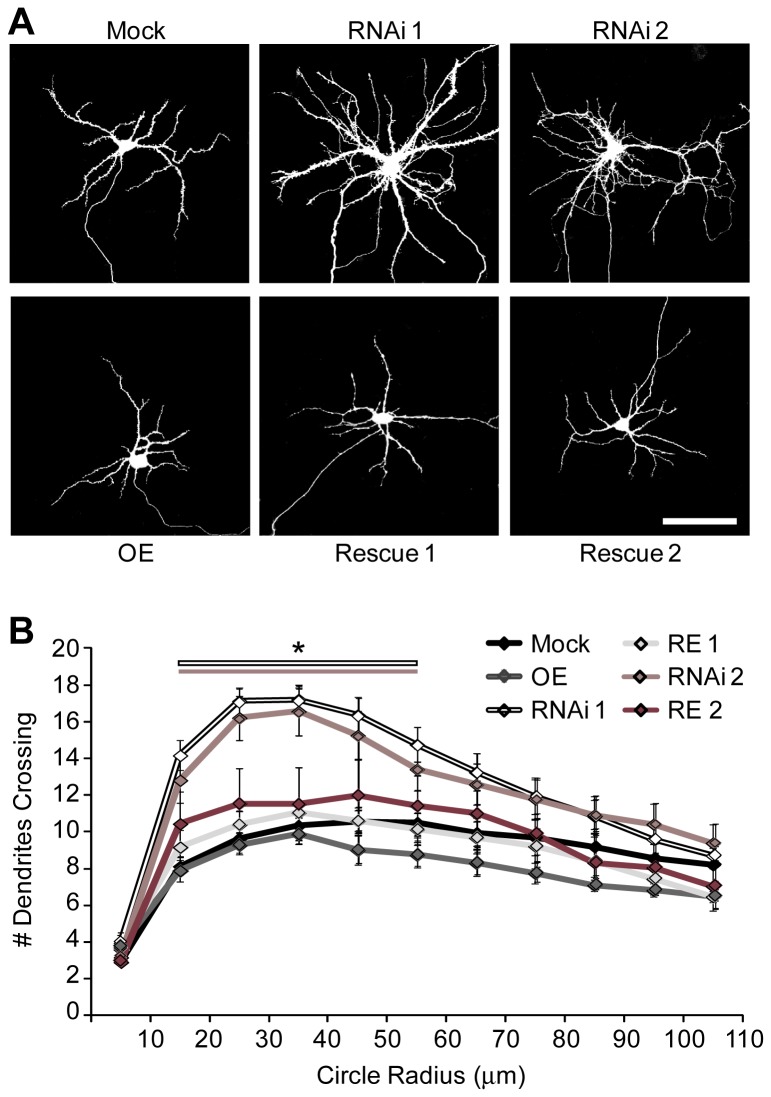
Rem2 is a negative regulator of dendritic complexity. **A**) Representative images of Mock, RNAi 1, RNAi 2, OE, RE 1, and RE 2 transfected neurons. Scale bar = 50 µM. **B**) Quantification of dendritic complexity for each condition using Sholl analysis. n > 40 neurons per condition. * p ≤ 0.05 compared to mock determined by multivariate ANOVA with a Tukey’s post hoc test.

Previous work in our lab identified Rem2 as a positive regulator of synapse development and a negative regulator of dendritic complexity in hippocampal neurons [15,24,25]. To assess the effect of Rem2 knockdown by RNAi 2 on dendritic complexity, we again transfected 4 DIV cultured hippocampal neurons with GFP and either Mock, RNAi 1, RNAi 2, OE, RE 1, and RE 2 as described above. At 14 DIV neurons were fixed and GFP-positive neurons were imaged to determine changes in dendritic complexity. Dendritic complexity was measured at 14 DIV by performing Sholl analysis [24,25,27], in which a series of concentric circles of increasing diameter, centered at the soma, are superimposed on a neuron image and the number of dendrites intersecting these circles is quantified. Neurons with decreased Rem2 expression, as a result of either RNAi 1 or RNAi 2, showed a significant increase in dendritic complexity between 20 and 60 µm from the soma (Figure 2A, B). This phenotype was effectively rescued with the RNAi-resistant cDNA (RE 1 and RE 2; Figure 2A, B). In agreement with previous work [25], we did not observe a Rem2 overexpression phenotype on dendritic complexity (Figure 2A, B).

**Figure 3:**
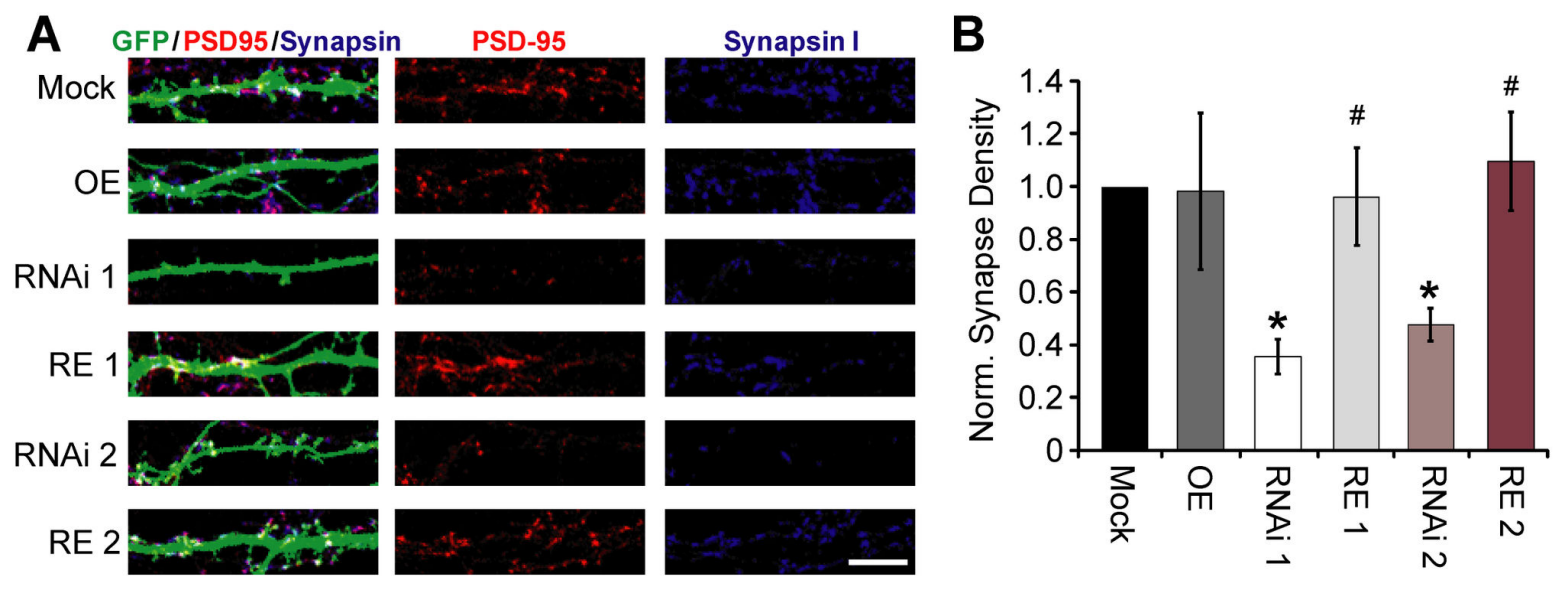
Rem2 is important for excitatory synapse formation in primary hippocampal neurons. **A**) Representative images of excitatory synapse staining for the presynaptic marker, synapsin I (blue) and the postsynaptic marker PSD-95 (red) in neurons co-transfected with GFP and either: Mock (empty pSuper vector), overexpression with the Rem2 RNAi-resistant cDNA (OE), RNAi with the Rem2 short hairpin 1 (RNAi 1), RNAi with a second Rem2 short hairpin (RNAi 2), or rescue of RNAi 1 (RE 1), or RNAi 2 (RE 2) with the Rem2 RNAi-resistant cDNA. Scale bar indicates 5 µm. **B**) Synapse density measured by the co-localization of synapsin I and PSD-95 on a GFP positive stretch of dendrite. n > 40 neurons per condition. * p ≤ 0.05 compared to mock or ^#^ p ≤ 0.05 compared to each appropriate RNAi condition (i.e. RE1 vs. RNAi 1) using a two-way between-effect ANOVA followed by a Tukey’s post hoc test.

Next, we asked if targeting of Rem2 by the RNAi 2 construct resulted in decreased excitatory synapse density similarly to that observed with RNAi 1 [25]. To accomplish this goal, we again transfected 4 DIV hippocampal neurons with GFP and either Mock, RNAi 1, RNAi 2, OE, RE 1, or RE 2. At 14 DIV, neurons were fixed and stained for synapsin I, a presynaptic vesicle-associated protein, and PSD-95, a scaffolding protein highly enriched at the postsynaptic side of excitatory synapses. We defined synapse density as the triple co-localization of GFP, synapsin I (presynaptic), and PSD-95 (postsynaptic), divided by the total GFP area (excluding the soma). Knockdown of Rem2 with RNAi 1 significantly decreased excitatory synapse density, a phenotype that was rescued by co-expression of the RNAi-resistant cDNA (RE 1; Figure 3A, B), as previously reported [25]. Similarly, knockdown with RNAi 2 also significantly reduced synapse density, and this phenotype could also be rescued by co-expression of an RNAi-resistant Rem2 cDNA (Figure 3A, B). Again, we did not observe an effect of Rem2 overexpression on synapse density (OE; Figure 3 A, B) [25]. Taken together, these results demonstrate that multiple Rem2-targeted shRNAs cause a similar decrease in excitatory synapse density and a similar increase in dendritic complexity, confirming that these phenotypes are the result of Rem2 knockdown and are not due to off-target effects of the shRNAs. Further, these results verify that endogenous Rem2 functions to promote excitatory synapse density and restrict dendritic complexity, as has been shown previously [15,25].

**Figure 4:**
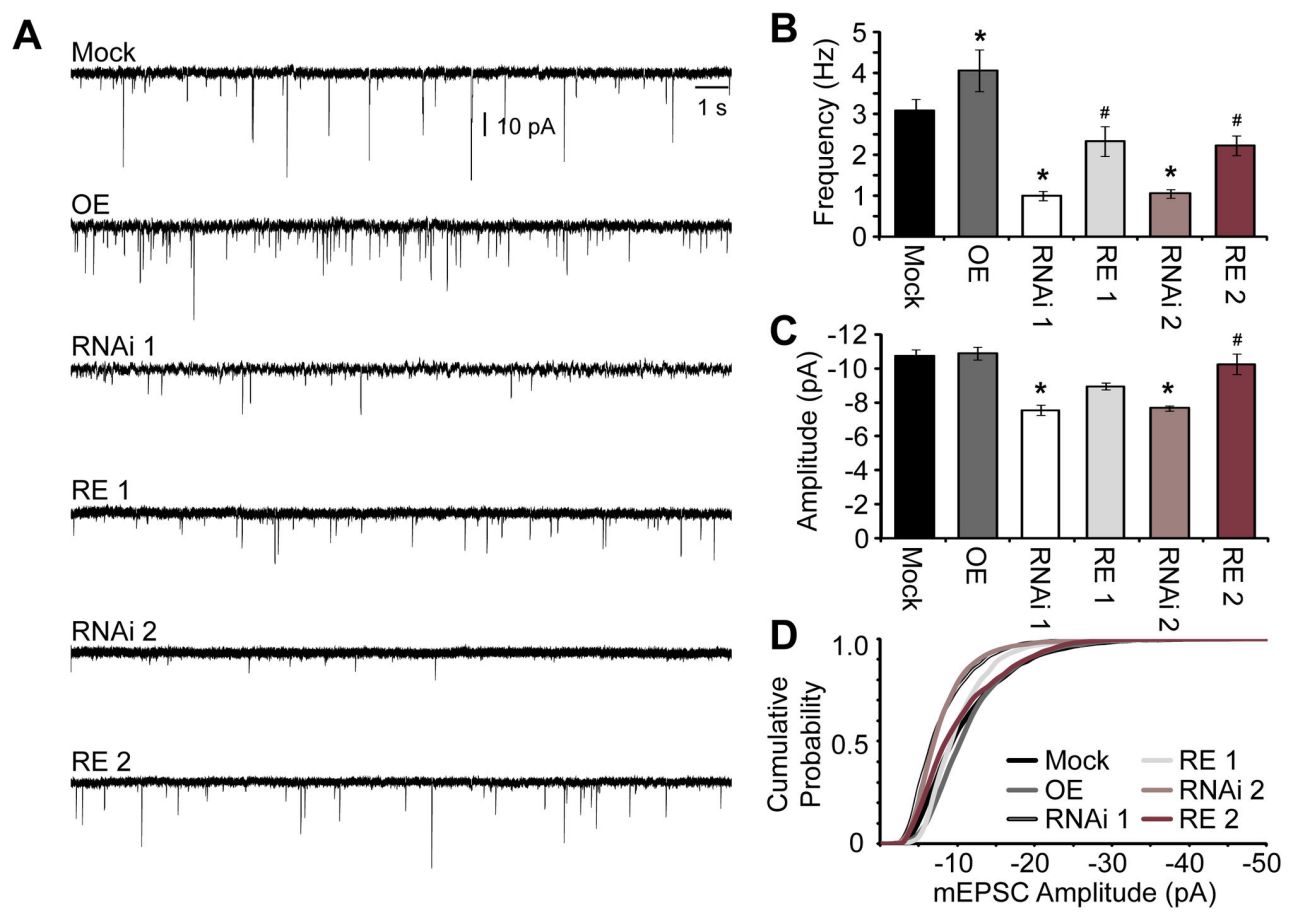
Rem2 is important for functional excitatory synapse formation. **A**) Representative 20 s miniature excitatory postsynaptic current (mEPSC) trace selected for each experimental condition: Mock, OE, RNAi 1, RE 1, RNAi 2, and RE 2, recorded from primary hippocampal neurons at 14 DIV. **B** and **C**) Average mEPSC frequency (**B**) and amplitude (**C**) measured for each condition for a total of 300 s. **B** and **C**) n ≥ 15 neurons per condition (3 separate experiments). **D**) Cumulative distribution of mEPSC amplitudes for Mock, OE, RNAi 1, RE 1, RNAi 2, and RE 2 generated from 100 random mEPSC per cell (n=15 cells per condition). RNAi 1 and RNAi 2 mEPSC amplitude plots were significantly different from Mock (p < 0.05, Kolmogorov-Smirnov test). Additionally, the leftward shift following RNAi 1 and RNAi 2 was significantly different from their respective rescue conditions (RE 1 and RE 2, respectively; p < 0.05). OE, RE 1, and RE 2 were not significantly different from Mock. (**B** and **C**) * p ≤ 0.05 compared to mock or ^#^ p ≤ 0.05 compared to the appropriate RNAi condition using a one-way ANOVA followed by a student’s independent *t*-test.

We next asked if the decreased excitatory synapse density that we observed by immunocytochemistry (Figure 3) reflected a change in functional synapse density as has been previously observed in hippocampal cultures following knockdown of Rem2 [15,18]. We co-transfected cultured hippocampal neurons at 4 DIV with either Mock, RNAi 1, RNAi 2, OE, RE 1, or RE 2. At 14-15 DIV whole-cell voltage clamp recordings were performed to measure spontaneous miniature excitatory postsynaptic currents (mEPSCs) in GFP-positive neurons. We found that knockdown of Rem2 with either short hairpin (RNAi 1 or RNAi 2) significantly reduced mEPSC frequency (Figure 4A, B, Mock: 3.09 ± 0.27 Hz, n=19; RNAi 1: 0.99 ± 0.12 Hz, n=15; and RNAi 2: 1.06 ± 0.1 Hz, n=19). These effects were rescued by co-transfection with the RNAi-resistant cDNA (Figure 4A, B, RE 1: 2.34 ± 0.36 Hz, n=15; RE 2: 2.23 ± 0.23 Hz, n=15), confirming that these phenotypes are the result of Rem2 knockdown. Additionally, knockdown with either short hairpin (RNAi 1 or RNAi 2) significantly reduced mEPSC amplitudes compared to Mock (Figure 4C, Mock: -10.72 ± 0.41 pA; RNAi 1: -7.54 ± 0.3 pA; and RNAi 2: -7.66 ± 0.14 pA). This effect was rescued for RNAi 2 (RE 2: -10.25 ± 0.58 pA), but not rescued for RNAi 1 (RE 1: -8.95 ± 0.21 pA), although we did observe a trend towards increased mEPSC amplitude in the RE 1 condition. We also observed a small but statistically significant increase in mEPSC frequency (OE: 4.07 ± 0.51 Hz, n=15) with no change in amplitude upon Rem2 overexpression in hippocampal neurons (OE: -10.89 ± 0.39 pA).

To gain a better understanding of the distribution of mEPSC amplitudes across conditions, we also analyzed the cumulative distribution of amplitudes for each of the six conditions (Figure 4D). Knockdown of Rem2 with either short hairpin (RNAi 1 and RNAi 2) led to a significant shift towards smaller amplitude events in the cumulative distribution of mEPSC amplitudes compared to Mock (p< 0.05 RNAi 1 vs Mock and RNAi 2 vs Mock, Kolmogorov-Smirnov test). This shift in distribution with RNAi was rescued (Figure 4D; p < 0.05 RNAi 1 vs RE 1 and RNAi 2 vs RE 2), with the two rescue conditions having distribution plots that are not significantly different from Mock. Taken together, these analyses suggest that Rem2 knockdown causes a decrease in mEPSC amplitude.

A decrease in overall synapse density as assayed by immunostaining (Figure 3) combined with a decrease in mEPSC frequency following Rem2 knockdown suggests an overall decrease in functional synaptic contacts. A change in mEPSC amplitude could be attributed to a change in the postsynaptic receptor density or individual receptor conductance [28,29]. While we did not observe a change in membrane capacitance, there was a significant increase in input resistance (R_*in*_) upon Rem2 knockdown using either shRNA (Table 1, RNAi 1 and RNAi 2). The underlying cause of a decrease in mEPSC amplitudes coupled with increased R_*in*_ in the absence of changes in *C*_*m*_ could be a decrease in the overall density of glutamate receptor-channels (which is a postsynaptic conductance) or other, unidentified changes in membrane conductances [28,29]. In summary, the physiological measurements of mEPSC amplitude and mEPSC frequency, combined with a decreased level of PSD-95 and synapsin I co-localization indicate a significant reduction in glutamatergic synaptic transmission in hippocampal neurons following Rem2 RNAi.

**Table 1:** Passive membrane properties.

Condition	N	R_*in*_	Capacitance
Mock	19	293 ± 7.7 MΩ	28.8 ± 1.1 pF
OE	16	281 ± 18 MΩ	25.5 ± 1.4 pF
RNAi 1	19	448 ± 22 MΩ*	35.0 ± 1.7 pF
RE 1	14	300 ± 22 MΩ^#^	40.2 ± 3.7 pF
RNAi 2	19	719 ± 40 MΩ*	41.4 ± 1.8 pF
RE 2	16	449 ± 17 MΩ*^#^	39.6 ± 2.1 pF

Change in passive membrane properties, input resistance (R_*in*_) and membrane capacitance, measured in 14 DIV neurons. These are the membrane properties for those cells reported in Figure 4. *p ≤ 0.05 compared to mock or ^#^p ≤ 0.05 compared to each appropriate RNAi condition with a one-way ANOVA followed by an independent student’s *t*-test. All other comparisons are not significant.

**Figure 5:**
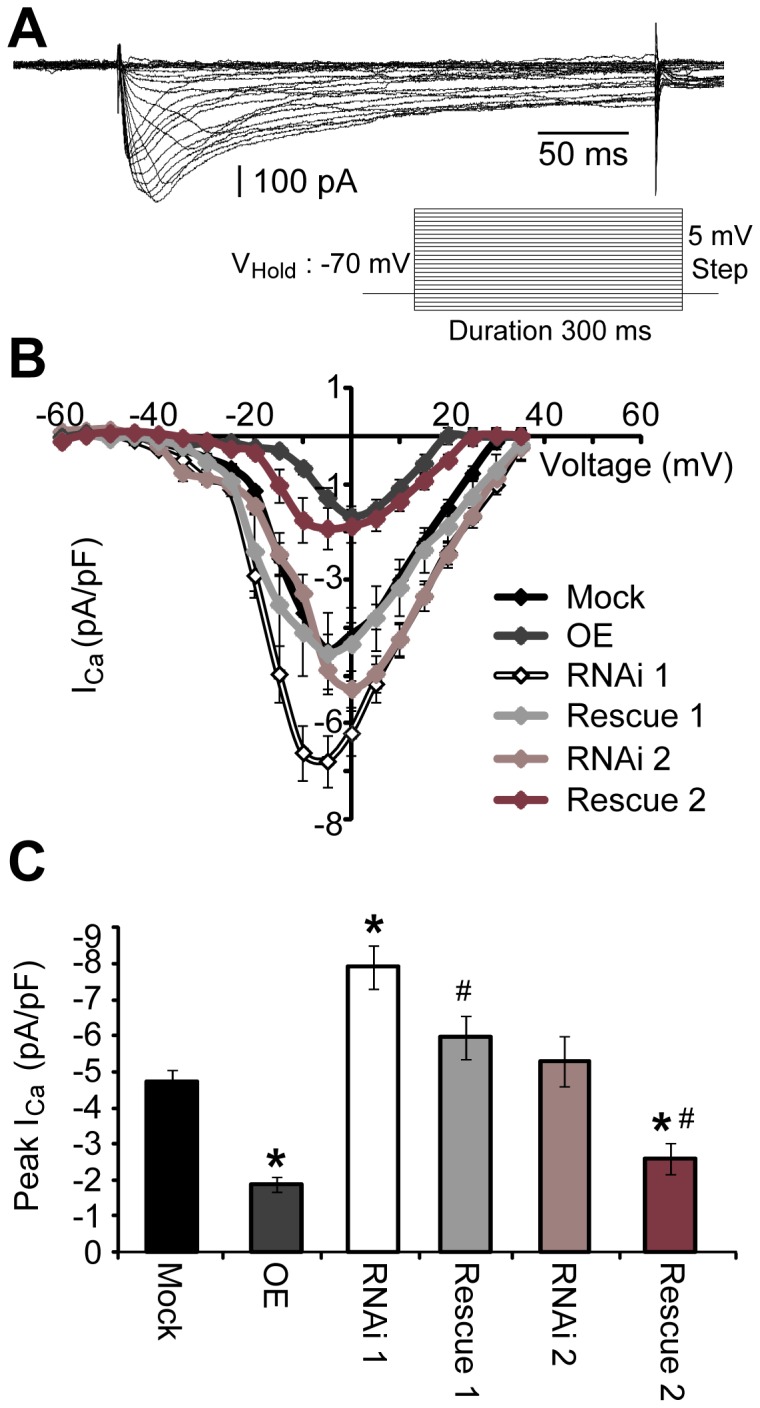
Rem2 overexpression significantly reduces voltage-gated calcium current. **A**) Cells were given a series of voltage steps (-90 to +40 mV) from a holding potential of -70 mV (inset). Representative isolated calcium current, following offline subtraction of a recording in the presence of nifedipine (10 µM). **B**) Current-voltage (I–V) relationship of calcium current density (pA/pF) following Rem2 knockdown, rescue, or overexpression. **C**) Peak calcium current amplitude was determined following a ramp step from -80 mV to +40 mV (duration 500 ms). n ≥ 10 cells per condition (3 separate experiments). * p ≤ 0.05 compared to mock or ^#^ p ≤ 0.05 compared to each appropriate RNAi condition determined using a one-way ANOVA followed by a student’s independent *t*-test.

Much of the interest in the RGK protein family is due to the observed suppression of voltage-gated calcium channel-mediated currents as a result of RGK family member overexpression in various cell types [20]. Specifically with respect to Rem2, overexpression of Rem2 significantly decreases voltage-gated calcium currents in heterologous cells [8,17], sensory ganglion neurons [16], and cultured hippocampal neurons [18]. We sought to determine if a function of endogenous Rem2 is to regulate VGCC by performing the complementary, loss-of-function experiment. We asked if decreasing Rem2 expression resulted in increased VGCC currents, as predicted based on the overexpression studies, in primary hippocampal neurons. Cultured hippocampal neurons were transfected at 4 DIV with GFP and either Mock, RNAi 1, RNAi 2, OE, RE 1, or RE 2, as described above. Whole-cell patch clamp recordings were performed on GFP-positive neurons at 8 DIV to examine the effect of Rem2 expression on VGCC currents. Calcium currents were recorded in the presence of tetrodotoxin (TTX; 1 µM; to block voltage-gated sodium channels), tetraethylammonium (TEA; 10 mM), 4-Aminopyridine (4-AP; 5 mM), and Cesium Chloride (CsCl; 5mM; all to block voltage-gated potassium channels), and ZD 7288 (to block I_H_ current) extracellularly. Voltage-gated calcium currents were elicited following a 300 ms pulse from -90 to + 40 mV in 5 mV steps from a holding potential of -70 mV (Figure 5A, inset) and confirmed for each transfection condition by applying the L-type VGCC antagonist Nifedipine (10 µM) to the bath (Figure 5A). A current-voltage (IV) relationship plot was calculated by taking the peak amplitude at each voltage step divided by the cells’ membrane capacitance (Figure 5B). Additionally, peak VGCC current density was calculated for each cell following a ramp step from -80 mV to + 40 mV for a duration of 500 ms (Figure 5C).

Compared to mock condition (Mock Peak I_Ca_ density: -4.72 ± 0.35 pA/pF; n=14), Rem2 OE significantly reduced voltage-gated calcium currents (OE Peak I_Ca_ density: -1.89 ± 0.2 pA/pF; n=13) as previously reported in neurons [16,18]. For cells transfected with RNAi 1, we observed a significant increase in VGCC current density (RNAi 1 Peak I_Ca_ density: -7.91 ± 0.6 pA/pF; n=14) that returned to mock levels following co-transfection with the Rem2 RNAi-resistant cDNA (RE 1 Peak I_Ca_ density: -5.96 ± 0.6 pA/pF; n=10; Figure 2B, C). To our surprise, we did not observe a significant change in the peak I_Ca_ density in cells transfected with RNAi 2 (RNAi 2: -5.3 ± 0.69 pA/pF; n=12), similar to previous reports [18]. In addition, we found that co-transfection of RNAi 2 with a Rem2 RNAi-resistant cDNA suppressed VGCC current (RE 2 Peak I_Ca_ density: -2.58 ± 0.43 pA/pF; n=10), similar to the phenotype observed with Rem2 overexpression. Because only one shRNA targeting Rem2 caused a decrease in I_Ca_ density in our study, it is unclear whether regulation of VGCC is an endogenous function of Rem2 in hippocampal neurons. We cannot exclude the possibility that the increase in I_Ca_ density observed with RNAi 1 is due to an off-target effect of RNAi 1 that specifically affects VGCCs. If that were to be the case, then the observed “rescue” of RNAi 1 with the RNAi-resistant cDNA would most likely be due to an additive effect of the decrease in I_Ca_ density observed with Rem2 overexpression coupled with an increase in I_Ca_ due to transfection of Rem2 RNAi 1 (Figure 5).

## Discussion

In this study, we sought to investigate the endogenous function of the RGK family member Rem2 in hippocampal neurons. In the absence of a mouse model in which the Rem2 gene has been deleted, the most straightforward approach to interfering with endogenous Rem2 expression is the use of RNAi technology. Thus, we designed an additional short hairpin RNAi construct, targeting an entirely independent sequence of the Rem2 mRNA, in order to compare cellular phenotypes obtained with this new shRNA against a previously characterized shRNA [25]. The benefit of comparing the phenotypic effects of multiple, independent shRNA sequences is that while both shRNAs target Rem2, they will most likely have entirely unique “off-target” RNAi effects (i.e. recognition of transcripts other than the intended target by the RNAi reagent) [30–32]. Thus, any phenotypes which manifest using both shRNAs are strongly correlated with knockdown of Rem2 expression rather than off-target effects of the RNAi technology.

This situation is analogous to traditional genetic approaches where multiple mutant alleles of a given gene increase confidence that mutation in that gene is responsible for the observed phenotype. In contrast, any phenotypes that are observed with only one shRNA, or that cannot be rescued by co-transfection of an RNAi-resistant cDNA, could be due to off-target effects of the RNAi technology. In addition, the “gold standard” for validation of an RNAi reagent is rescue by co-transfection with an RNAi-resistant coding region of the targeted gene [30,32–34]. Thus, the accuracy of our results derives from our two-pronged approach to validation of RNAi-mediated Rem2 knockdown: the use of two shRNA reagents that are unrelated at the sequence level and rescue by co-transfection of each shRNA individually with an RNAi-resistant Rem2 cDNA. Any cellular phenotype obtained by Rem2 RNAi that does not meet these strict standards cannot be assigned to Rem2 function at this time.

Using each shRNA individually, we analyzed the effect of Rem2 knockdown on dendritic morphology, functional glutamatergic synapse formation, and voltage-gated calcium currents: three biological processes that have been reported to involve Rem2 function [8,15,16,18,24,25,35]. Our results demonstrate that Rem2 is in fact involved in at least two of these three processes. First, Rem2 is a negative regulator of dendritic complexity, as Rem2 knockdown with either shRNA results in a significant increase in dendritic complexity (Figure 2) that can be rescued by co-transfection of a Rem2 RNAi-resistant cDNA. Second, Rem2 is a positive regulator of functional excitatory synapse development, as loss of Rem2 expression using either shRNA results in a significant decrease in synapse density as assayed by immunocytochemistry (Figure 3) and mEPSC frequency and amplitude (Figure 4), effects which can also be rescued. Therefore, we can definitively conclude that endogenous Rem2 regulates those particular processes in neurons, and importantly, validates two independent Rem2-targeted shRNAs as useful tools for future investigation of Rem2 function in neurons.

Finally, while overexpression of RGK proteins has been shown to powerfully inhibit voltage-gated calcium channels in a number of systems [20], very few studies have addressed the effect of RGK family knockdown on VGCC function [18]. We found that while overexpression of Rem2 leads to a significant decrease in calcium current amplitude as observed previously (Figure 5) [8,16,18], Rem2 knockdown with only one of our shRNA constructs resulted in increased calcium current amplitude in hippocampal neurons. Thus the observed increase in VGCC current amplitude could be due to off-target effects of RNAi 1. This result is in agreement with a previous study in which no effect on VGCC currents was observed upon Rem2 knockdown [18]. Alternatively, subtle differences in the efficacy of Rem2 knockdown with each shRNA could also account for the disparate VGCC phenotypes. If that were to be the case, the differences in Rem2 knockdown efficacy with each shRNA are below the level of detection of either our immunoblotting or immunocytochemistry assays (Figure 1). Interestingly, we also observed differences in the neurons’ input resistance (R_*in*_) between RNAi 1 and RNAi 2 conditions. RNAi-mediated knockdown of Rem2 using shRNA 2 (i.e. RNAi 2) had increased R_*in*_ nearly two-fold compared to Mock (see Table 1). This change in R_*in*_, which influences how much a cell can depolarize in response to steady current, could therefore be masking an increase in VGCC current amplitude in RNAi 2 neurons. These caveats make it difficult to definitively conclude that the observed effect with RNAi 1 (i.e. significant increase in VGCC current amplitude) is an off-target effect of the RNAi reagent. Nonetheless, the most straightforward interpretation of our results is that endogenous Rem2 does not mediate calcium influx through VGCCs.

Interestingly, a Rem2 mutant unable to bind VGCCs was able to successfully rescue Rem2 RNAi-induced changes in both synapse density and dendritic complexity [25] indicating that the function of Rem2 in regulating synapse and dendrite development is independent of any direct regulation of VGCCs. Further support of this interpretation comes from a study by Wang et al. (2011) in which the authors also observed that Rem2 knockdown decreases mEPSC frequency but has no effect on calcium currents. However, the authors reach a somewhat surprising conclusion from this result. They interpreted the failure to observe an increase in calcium current density with Rem2 knockdown as an indication that other phenotypes observed with their Rem2 RNAi constructs (i.e. the decrease in mEPSC frequency) were due to off-target effects of the RNAi. This conclusion is based on two pieces of data: 1) a failure to rescue the mEPSC frequency with a GFP-tagged, RNAi-resistant Rem2 cDNA, and 2) a failure to observe a decrease in Rem2 levels with immunocytochemistry. The most likely explanation for the inability of the Wang et al. (2011) study to rescue the decrease in mEPSC frequency is that their GFP-tagged Rem2 cDNA does not have equivalent function to wildtype Rem2. All previous studies [12,21,25] observe Rem2 migrating as a doublet by Western blotting, while the GFP-tagged Rem2 used in the Wang study migrates as a single band suggesting that it may not be properly modified in the cell. Importantly, we have demonstrated that the slower migrating Rem2 band is the result of phosphorylation and that this post-translational modification is critical to Rem2 function [24]. A more direct approach to resolve the issue of the failure of the GFP-tagged, RNAi-resistant Rem2 cDNA to rescue would be to test the functionality of this construct in our experimental paradigm. However, our loss-of-function approach using a second validated shRNA targeting Rem2, eclipses any technical differences between previous studies [18,25] and strongly suggests that the endogenous function of Rem2 is to promote excitatory synapse formation and inhibit dendritic complexity.

In addition, the Wang et al. (2011) study used a pool of three shRNAs transfected simultaneously to knock down Rem2 expression levels; they then try to rescue the pool of shRNAs by co-transfection with the GFP-tagged Rem2. What distinguishes our study is that we utilize two discrete shRNAs transfected individually into neurons, each of which produce changes in functional synapse density and dendritic morphology, that are rescued by co-transfection with an RNAi-resistant Rem2 cDNA. Further, the commercial antibody used by Wang et al. to examine Rem2 knockdown in their cultures has never been validated, unlike our homemade antibody that has been proven to be specific for Rem2 both by Western blotting (Figure 1 this study [24,25]) and by immunocytochemistry (Figure 1 [25]). Another indication of the non-specificity of the commercial antibody used in the Wang et al. study comes from its failure to detect nuclear Rem2 that has been observed by us and others [25,36]. Unfortunately, we were unable to obtain the commercial antibody for direct comparison with our antibody due to its unavailability from the vendor (Santa Cruz: out of stock since November 2011).

While our current work agrees with the data presented in the Wang et al. 2011 study which shows that overexpression of Rem2 inhibits calcium influx through VGCCs in hippocampal neurons, neither our results nor those of Wang et al. (2011) demonstrate a clear effect on calcium currents in the absence of Rem2. Thus, our current model for Rem2 function is that phosphorylation of Rem2 by CaMKII causes Rem2 to become localized to the nucleus and this event is critical for Rem2 inhibition of dendrite morphology [24]. Further, Rem2 promotes the formation of functional glutamatergic synapses through a signaling mechanism that remains to be elucidated. Interestingly, a mutant Rem2 protein that is unable to bind Calmodulin retains its ability to promote excitatory synapse formation [25], suggesting that Rem2 signaling pathways distinct from those regulating dendritic morphology are required for excitatory synapse formation.
